# Stable and fluctuating temperature effects on the development rate and survival of two malaria vectors, *Anopheles arabiensis* and *Anopheles funestus*

**DOI:** 10.1186/1756-3305-6-104

**Published:** 2013-04-16

**Authors:** Candice L Lyons, Maureen Coetzee, Steven L Chown

**Affiliations:** 1 Centre for Invasion Biology, Department of Botany and Zoology, Stellenbosch University, Private Bag X1, Matieland, 7602, South Africa; 2 Malaria Entomology Research Unit, School of Pathology, Faculty of Health Sciences, University of the Witwatersrand, Johannesburg, South Africa; 3 School of Biological Sciences, Monash University, Victoria, 3800, Australia

**Keywords:** Constant *vs.* fluctuating temperatures, EIR, LDT, SET, *Anopheles*, Climate change

## Abstract

**Background:**

Understanding the biology of malaria vector mosquitoes is crucial to understanding many aspects of the disease, including control and future outcomes. The development rates and survival of two Afrotropical malaria vectors, *Anopheles arabiensis* and *Anopheles funestus*, are investigated here under conditions of constant and fluctuating temperatures. These data can provide a good starting point for modelling population level consequences of temperature change associated with climate change. For comparative purposes, these data were considered explicitly in the context of those available for the third African malaria vector, *Anopheles gambiae*.

**Methods:**

Twenty five replicates of 20–30 eggs were placed at nine constant and two fluctuating temperatures for development rate experiments and survival estimates. Various developmental parameters were estimated from the data, using standard approaches.

**Results:**

Lower development threshold (LDT) for both species was estimated at 13-14°C. *Anopheles arabiensis* developed consistently faster than *An*. *funestus*. Optimum temperature (T_opt_) and development rate at this temperature (μ_max_) differed significantly between species for overall development and larval development. However, T_opt_ and μ_max_ for pupal development did not differ significantly between species. Development rate and survival of *An*. *funestus* was negatively influenced by fluctuating temperatures. By contrast, development rate of *An*. *arabiensis* at fluctuating temperatures either did not differ from constant temperatures or was significantly faster. Survival of this species declined by *c*. 10% at the 15°C to 35°C fluctuating temperature regime, but was not significantly different between the constant 25°C and the fluctuating 20°C to 30°C treatment. By comparison, previous data for *An*. *gambiae* indicated fastest development at a constant temperature of 28°C and highest survival at 24°C.

**Conclusions:**

The three most important African malaria vectors all differ significantly in development rates and survival under different temperature treatments, in keeping with known distribution data, though differences among M and S molecular forms of *An*. *gambiae* likely complicate the picture. Increasing temperatures associated with climate change favour all three species, but fluctuations in temperatures are detrimental to *An*. *funestus* and may also be for *An*. *gambiae*. This may have significant implications for disease burden in areas where each species is the main malaria vector.

## Background

Malaria is Africa’s most significant vector-borne disease, accounting for over 200 million clinical cases and well over half a million deaths per year [1]. Although several factors affect malaria prevalence, including the efficacy of control interventions, it depends significantly on the entomological inoculation rate (EIR): the average number of infectious mosquito bites one person receives in a year [2]. The EIR is, in turn, dependent on the human biting rate, which is a product of the number of mosquitoes per human and the number of bites per mosquito. The number of mosquitoes in a population depends on the number of adults entering and leaving the population [3-5], both of which are affected significantly by environmental temperature. Low temperatures tend to limit aquatic stage development and adult activity of some *Anopheles* species, while extremely high temperatures lead to substantial mortality [6-8]. In the intermediate temperature range, development rate, feeding rate and adult survival increase with temperature, as is true of most ectotherms [9], often leading, in the case of vector-borne disease, to an increase in disease prevalence [3].

Given these relatively straightforward relationships between temperature and significant population parameters [3,5,10], it is perhaps not surprising that forecasts of increasing malaria burden with climate change have been made [11,12]. However, such forecasts are controversial for several reasons. First, despite claims that overall the disease burden will increase, several analyses have suggested that in some areas incidence will decrease and in others increase, leading to overall stasis (e.g. [13-15]) or even an overall observed range contraction in regions of stable malaria transmission [16]. When coupled with human intervention, the outcome in many regions should be a decline in disease prevalence. Second, much of the focus has been on changes in mean annual temperature. However, climate change involves more than a change in mean temperatures. Rather, extremes are changing too, with extreme high temperatures being more common than in the past [17]. Moreover, fluctuating temperatures can result in substantially different likelihoods of malaria transmission than constant temperatures [18,19], and the predicted temperature for optimal transmission has also been estimated at lower than previously thought [20]. In consequence, much attention is now being given to developing spatially accurate and biologically more realistic forecasts of changes in malaria prevalence [4,21,22], reflecting a general trend in the field of climate change impact forecasting for vectors and other species [23-25].

Mechanistic models [26,27] provide a useful means to forecast changes in malaria prevalence and can include significant nuances, such as the likely influence of evolutionary change and variation among species, populations and genotypes [26,28]. Nonetheless, they are dependent on the availability of basic physiological data, such as thermal responses, which, though relatively straightforward to collect, are often missing for vectors. Whilst much information is available on temperature effects on major life cycle components of the African vector *Anopheles gambiae*[7,29-31], much less is known about the thermal biology of the other two major vectors, *An*. *arabiensis* and *An*. *funestus*[32]. Indeed, one of the most comprehensive recent modeling approaches has highlighted the need for such data for these species [4]. These species are especially important in south-eastern Africa, an area for which environmental niche models suggest an increase in disease prevalence with climate change [5,12]. Although recent work has provided comprehensive information on extreme tolerance limits for *An*. *arabiensis* and *An*. *funestus*[8], the effects of temperature on development and intrinsic survival from egg to adult, have not been as comprehensively investigated (though see [29,33]). Furthermore, the influence of fluctuations in temperature on development, have not been extensively examined for African malaria vectors, despite the fact that fluctuating temperatures clearly influence other aspects of malaria transmission [18,19], and have long been known to affect anopheline development rate [34,35], as is the case for other insects [36].

This study examined the effects of constant and fluctuating temperatures on the development and survival of the two malaria vectors, *An*. *arabiensis* and *An*. *funestus*, while also making explicit comparisons with data collected elsewhere for *An*. *gambiae*. The results contribute to the information that is required for mechanistic forecasts of likely changes in mosquito population density, and therefore, ultimately, provide experimental data for estimating EIR associated with the change in climate that is taking place across southern Africa, and which is forecast to be substantial in the future [37-40].

## Methods

### Colony maintenance and egg collection

Eggs were collected from two laboratory colonies: the KGB-strain of *Anopheles arabiensis* originally established from individuals collected in Zimbabwe in 1975; and the FUMOZ-strain of *An*. *funestus* originating from individuals collected in Mozambique in 2000 [41]. Although the colonies have shown some laboratory adaptation in thermal responses, these have typically not been pronounced [8].

Colonies are routinely maintained at the insectary temperature of 25°C (± 2°C) and relative humidity of 80% (checked with a Masons thermohygrometer, Brannan, UK), with a 12:12 light/dark cycle and 30 min dusk/dawn simulation. The adults used in these experiments were provided with a 10% sugar water solution ad libitum and females were provided with a blood meal every alternative day. *Anopheles arabiensis* usually requires at least two blood meals to produce eggs, while *An*. *funestus* requires at least three [42]. Hence, only females that had received at least three blood meals were used for egg collections. Female mosquitoes of each colony were given no longer than half of one dark cycle (6 h) in which to lay eggs in the provided egg-plates (darkened plastic petri dishes 70 mm diameter filled with distilled water). This 6 h period was chosen to allow the chorion of the mosquito eggs to harden before being disturbed (see [42]). Following the 6 h period, eggs from each species were separated into 200 ml bowls (filled with distilled water) with between 20 to 30 eggs per bowl. Twenty-five bowls were set up per species. These 25 replicates were the basic sample unit used for assessment of development rate at each of several temperatures (i.e. n=25 per temperature): constant temperatures of 15, 18, 20, 22, 25, 28, 30, 32 and 35°C; and two fluctuating temperature regimes: 15°C to 35°C, and 20°C to 30°C, each with a mean temperature of 25°C, and the lowest temperature set for the 12 hour scotophase of a 12L:12D cycle. These temperatures were chosen to represent those within which development to adulthood is known to occur in other *Anopheles* species (e.g. [7,29]). Temperatures were maintained to within ±0.5°C through the use of PTC-1 Peltier portable temperature control cabinets (Sable Systems, Las Vegas, Nevada, USA) or through the use of an incubator (SANYO, MIR-154, SANYO Electric Co. Ltd., Osaka, Japan) and were checked using a mercury thermometer. The photoperiod was maintained through non-heating fluorescent tubes connected to a timer. Eggs were maintained under these conditions and larvae reared to eclosion. To prevent eggs from sticking to the sides of replicate bowls, they were washed down using distilled water of the same temperature as each relevant treatment. Larval food comprised a mixture of finely ground dog biscuits and yeast extract. Larvae were fed once or twice daily depending on instar, and adults were killed following eclosion.

### Development rate

All temperature treatments were checked for any developmental change every 8–12 hours depending on stage of development. The positions of replicates were randomized in the incubators. The length of time that 50% of the population in each replicate took to reach each life stage, and total time to adulthood (again 50% of the population) was recorded for each of the 25 replicates per temperature treatment and for each species. The 50% criterion was used because of several substantial outliers, which could not be distinguished as the outcome of delayed egg hatch [43] or experimental artefact, and were therefore given less weight using this procedure. Rate-temperature curves were plotted for each species using 1/mean time (days^-1^) to larva/pupa/adult emergence per temperature. Using the linear part of the curve for each species (between 15°C and 32°C for *An*. *arabiensis* and between 15°C and 30°C for *An*. *funestus*), ordinary least squares linear regression as implemented in R (v. 2.15.1) (R Foundation for Statistical Computing, Vienna, Austria) was used to estimate the lower developmental threshold (LDT: -slope/intercept in °C) and the sum of effective temperatures (SET: 1/slope in degree-days) for each life stage change (i.e. egg, larva, pupa), and for overall development from egg to adult [44-46]. To compare overall development rates between the two fluctuating temperature treatments and their constant mean of 25°C, an analysis of variance (ANOVA) was used (R v. 2.15.1) for each species. Normality and homogeneity of variance were first checked using Shapiro-Wilk’s and Levene’s tests, respectively (Additional file 1). In some cases deviations from normality were observed, but generally, few deviations occurred and the model assumptions were met, allowing use of a parametric ANOVA which is reasonably robust and insensitive to deviations from normality, provided designs are balanced [47]. Mean development time in days for each stage and overall across all 11 temperature treatments are shown in Additional file 2. To compare development rates of each stage and for overall development between species, general linear models were implemented in R (v. 2.15.1) for each stage comparison and overall egg to adult development using temperature and species as categorical predictors in the model and development rates as response variables. Deviations from normality occurred in some instances, but model assumptions were generally met [47,48] (Additional files 3 and 4).

To determine the optimum development temperature (T_opt_) and the maximum development rate associated with this temperature (μ_max_) (see [49]), a non-linear curve-fitting approach was adopted using TableCurve 2D (v. 5.01, SYSTAT Software Inc., 2002, San Jose, California, USA) (Additional files 5, 6, 7) (see [49]). T_opt_ and μ_max_ were determined from the equations for the best fit curve, which differed among stages and between species (Table 1, Additional files 8 and 9). To compare T_opt_ and μ_max_ of *An*. *arabiensis* to that of *An*. *funestus*, one replicate for each temperature treatment was selected at random (without replacement) to provide 25 separate curves for overall development rate for each species and for each life stage. The equations used to obtain T_opt_ and μ_max_ for overall development and development of each stage across all 25 replicates are presented in Additional file 10. Except in a few cases (pupal development rates) these equations all had r^2^ values above 0.90. The same equations for all 25 replicates were chosen to minimize discrepancies when comparing T_opt_ and μ_max_ between species. T_opt_ and μ_max_ were then compared, for overall development and for each life stage, between the species using t-tests (R v. 2.15.1, R Foundation for Statistical Computing, Vienna, Austria).

**Table 1:** **Developmental parameters for each life stage and overall**, **for *Anopheles arabiensis *and *Anopheles funestus***

**Species**	**Life stage**	**T**_**opt**_ (°**C**)	μ_**max**_ (**days**^-**1**^)	**LDT** (°**C**)	**SET** (**DD**)
*An*. *arabiensis*	Eggs	31.1	0.7727	13.1	25.4
	Larvae	31.1	0.208	14.3	75.8
	Pupae	28.7	1.6109	14	13.8
	Overall	31.8	0.1286	13.4	137
*An*. *funestus*	Eggs	31	0.5772	12.7	35.6
	Larvae	30.9	0.1357	13.8	116.3
	Pupae	27.3	0.9052	14.4	16.3
	Overall	31.1	0.0813	14	166.7

Optimum temperature (T_opt_), development rate at the optimum temperature (μ_max_), lower developmental threshold (LDT) and sum of effective temperatures (SET in degree-days (DD)) for eggs, larvae, pupae and the overall development of *Anopheles arabiensis* and *Anopheles funestus*.

### Survival

Although development rate generally increases with increasing temperature up to the optimum [50,51], high development rates are often accompanied by mortality and reduced population output [7,50,52]. In consequence, overall survival from egg to adult was recorded as the proportion of eggs that emerged as adults (expressed as a percentage). This % survival was recorded for all 25 replicates per temperature treatment. To assess differences in survival between the fluctuating temperature treatments and their constant mean (25°C), a generalized linear model with a binomial distribution of errors and logit link function was used (R v. 2.15.1). To illustrate the effect of temperature on survival of each species, mean percentage survival (± standard error) was plotted at each constant temperature and in a comparison between the two fluctuating temperatures and constant mean of 25°C.

Comparisons between the species examined here and the third major African malaria vector, *Anopheles gambiae s*.*s*., were made using data previously gathered for this species [7]. Although this previous work was conducted under different experimental conditions [7], a comparison between development rates and survival of the three species under a range of temperatures is still warranted given that food limitation did not occur and that temperature effects are likely to be the most profound given experimental treatments in both studies.

## Results

### Development rate

**Figure 1:**
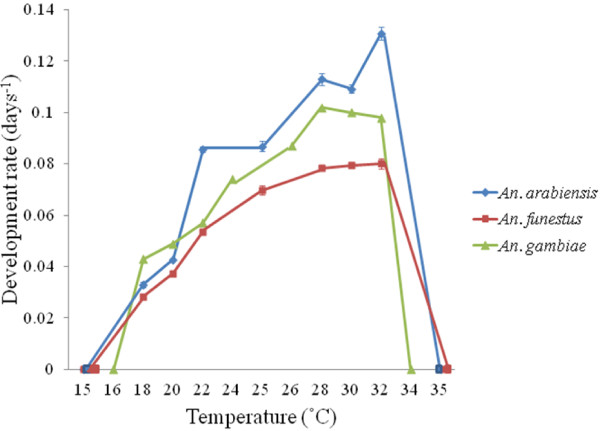
**Mean development rate and constant temperature.** Mean development rate (days^-1^) per constant temperature (ranging from 15°C to 35°C) for *Anopheles arabiensis* (blue ♦), *An*. *funestus* (red ■) and *An*. *gambiae* (green ▲) (data for *An*. *gambiae* obtained from [7]). Lines linking data points are not fitted and are for reference only. 95% confidence intervals are shown for *An*. *arabiensis* and *An*. *funestus*, but are typically obscured by the data points. For the full range of temperatures, the development rate of each species is typically non-linear. For each species, there exists a linear part to this curve, which differs between species.

**Figure 2:**
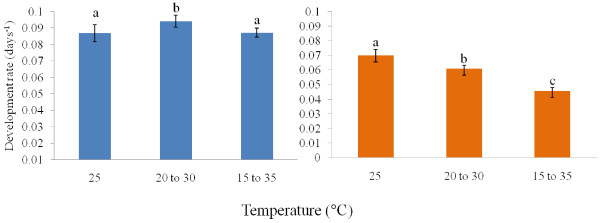
**Development rate at constant and fluctuating temperatures.** Development rate (days^-1^) of *Anopheles arabiensis* (left) and *Anopheles funestus* (right) at the two fluctuating temperature regimes and the constant mean of 25°C. Differences in lower case letters indicate significant differences in development rates (within each species) between the two fluctuating temperature regimes of 20 to 30°C and 15 to 35°C, and their constant mean of 25°C (ANOVA: *An*. *arabiensis* df=2, 72, F=25.5, P<0.0001; *An*. *funestus* df=2, 72, F=395.3, P<0.001). Development at 25°C was significantly faster than at fluctuating temperatures for *An*. *funestus* but did not differ markedly between treatments for *An*. *arabiensis*.

Total development rate from egg to adult of *An*. *arabiensis* and *An*. *funestus* increased between 18°C and 32°C and between 18°C and 30°C, respectively, in a linear fashion (Figure 1). At 15°C and 35°C, no development from egg to adult occurred in either species (Figure 1). Although, experimentally, no development occurred at 15°C and 35°C, lower developmental thresholds for *An*. *arabiensis* and *An*. *funestus* were estimated as ~13°C and ~14°C, respectively (Table 1). Development rates of each stage, and from egg to adult across all temperatures differed significantly between species, with *An*. *arabiensis* showing consistently faster development rates than *An*. *funestus* (Table 2). Moreover, the significant temperature*species interaction indicated a steeper slope of the rate-temperature relationship in *An*. *arabiensis* (Figure 1) than in *An*. *funestus*, reflected in the lower SET value for the former species (Table 1). Species comparisons of μ_max_ and T_opt_ likewise revealed significantly higher T_opt_ and μ_max_ for overall development and for larval development in *An*. *arabiensis* than in *An*. *funestus* (Tables 1, 3), but no significant difference in T_opt_ or μ_max_ for pupal development, and only significantly different μ_max_ for egg development (Table 3). Development rate of *An*. *arabiensis* at 25°C did not differ significantly from development rate at 15°C to 35°C, although development rate at 20°C to 30°C was significantly faster than both of these (Figure 2). In contrast, in *An*. *funestus*, fluctuating temperatures led to significantly slower development rates than at 25°C (Figure 2). For both species, T_opt_ was higher for eggs and larvae than it was for pupae (Table 1).

**Table 2:** Effects of species and temperature on development rates of each life stage and overall development

**Stage**	**Predictor**	**df**	**SS**	**F**	**P**-**value**
Eggs	Temperature	8	2.63	80.39	< 0.0001
F_17,432_=184.8; P<0.0005	Species	1	0.86	209.45	< 0.0001
	Temperature*Species	8	1.51	46.29	< 0.0001
Larvae	Temperature	8	0.24	213.33	< 0.0001
F_17,432_=460.5; P<0.0005	Species	1	0.03	234.73	< 0.0001
	Temperature*Species	8	0.05	52.12	< 0.0001
Pupae	Temperature	8	8.29	8.07	< 0.0001
F_17,432_=20.51; P<0.0005	Species	1	7.75	60.34	< 0.0001
	Temperature*Species	8	8.62	8.39	< 0.0001
Egg to adult	Temperature	8	0.08	361.88	< 0.0001
F_17,432_=795.9; P<0.0005	Species	1	0.02	766.33	< 0.0001
	Temperature*Species	8	0.02	110.25	< 0.0001

Results are from general linear models comparing development rates (days^-1^) of each stage and from egg to adult, between species, as a function of temperature and species. Model results are shown in the left hand column under each stage comparison.

**Table 3:** **Comparing optimum temperatures and development rates at these temperatures**, **between *Anopheles arabiensis *and *Anopheles funestus***

**Life stage**	**t**-**value**	**df**	**P**-**value**
**T**_**opt**_			
Eggs	−0.06	48	0.9492
Larvae	−2.03	48	0.0475
Pupae	−1.86	48	0.0694
Overall	−3.97	48	0.0002
μ_**max**_			
Eggs	−16.34	48	< 0.0001
Larvae	−18.86	48	< 0.0001
Pupae	−1.98	48	0.0537
Overall	−33.71	48	< 0.0001

Results from two-sample t-tests comparing optimum temperature (T_opt_) and rate of development at the optimum temperature (μ_max_) of each stage between *Anopheles arabiensis* and *Anopheles funestus*.

### Survival

**Figure 3:**
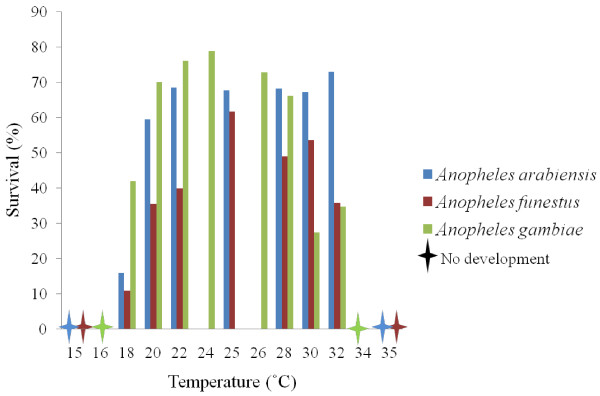
**Mean survival at constant temperatures.** Mean percentage survival per constant temperature for *Anopheles arabiensis* (blue), *An*. *funestus* (red) and *An*. *gambiae* (green) (data for *An*. *gambiae* obtained from [7]). Error bars are shown for *An*. *arabiensis* and *An*. *funestus*. Survival of *An*. *gambiae* is highest at the lower end of the temperature range, while survival of *An*. *arabiensis* is highest towards the upper end of this temperature scale. *An*. *funestus* displays lower survival at all temperatures when compared to the other two vector species. No development and hence, no survival occurred at 15°C and 35°C for *An*. *funestus* and *An*. *arabiensis*, while *An*. *gambiae* did not develop at 16°C and 34°C.

**Figure 4:**
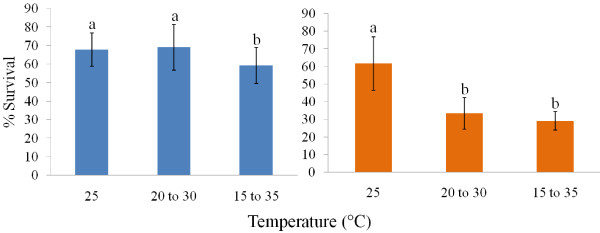
**Mean survival at constant and fluctuating temperatures.** Mean percentage survival for *Anopheles arabiensis* (left) and *Anopheles funestus* (right) between the two fluctuating temperature regimes and constant mean of 25°C. Differences in lower case letters indicate significant differences in survival between temperature treatments within each species (GLZ with binomial distribution and logit link: *An*. *arabiensis* df=2, 72, chi-squared=15.4, P<0.001; *An*. *funestus* df=2, 72, chi-squared=164.9, P<0.0001). Survival of *An*. *arabiensis* was only negatively affected at the most variable temperature treatment. *Anopheles funestus* experienced severely lowered survival at the two fluctuating temperature treatments when compared to the constant 25°C treatment.

Survival (%) from egg to adult was highest at 32°C for *An*. *arabiensis* and at 25°C for *An*. *funestus* (Figure 3). Complete mortality was found at 15°C and 35°C for both *An*. *arabiensis* and *An*. *funestus* (Figure 3). In the case of *An*. *arabiensis*, only the 15°C to 35°C fluctuating temperature led to a significant decline in survival by comparison with the constant 25°C conditions (Figure 4), and then only by *c*. 10%. In contrast, in *An*. *funestus* survival was lower in both sets of fluctuating temperature conditions compared with the constant 25°C, and the reduction in survival was by at least half (Figure 4).

## Discussion

Of the three major African malaria vector species, *An*. *arabiensis* had the fastest overall development rate over a wide range of temperatures. The faster rate by comparison with *An*. *gambiae* (illustrated in Figure 1) contrasts with that of at least two previous studies [29,33]. The contrasting outcomes may reflect genotypic differences among the populations used that may have significant effects on a range of traits [53]. Alternatively, the *An*. *gambiae* study reared larvae at different densities and under different feeding regimes [7] than the present study which may have influenced the development response of this species to temperature. Nonetheless, both species showed temperature optima for development at *c*. 32°C, although the range of temperatures over which development rate is fastest is broader in *An*. *gambiae* (28-32°C, [7]), than it is in *An*. *arabiensis* (32°C). This difference in performance breadth is in keeping with a slightly greater niche width calculated for and generally wider habitat use in *An*. *gambiae* than *An*. *arabiensis*[53-55], though the significance of the M and S molecular forms of *An*. *gambiae*[56] in influencing these patterns is not clear. In comparison, *An*. *funestus* had a much longer development time than both of these species, with a similar optimum temperature for development, in keeping with its preference for cooler, more permanent and often shaded habitats [53-55].

Temperature also affected survival differently in the three species (see also [8,30,35,57]). Peak survival was highest at 32°C in *An*. *arabiensis*, though survival rates were similar between 22°C and 32°C. In *An*. *gambiae* survival peaks at 24°C, and is fairly similar between 22°C and 28°C [30], dropping rapidly above 30°C. These differences in survival rate at different temperatures between *An*. *gambiae* and *An*. *arabiensis*[29] contrast strongly with the situation in *An*. *funestus*. A single fairly pronounced survival optimum occurred at 25°C, with substantial declines on either side of this temperature.

The presumably typical exposure of *An*. *arabiensis* to fluctuating temperatures, given that it tends to prefer smaller water bodies than does *An*. *funestus*[54,55,58] and the greater variability in temperature of smaller ponds [59,60], appears to be reflected in the responses to fluctuating temperatures of development rate and survival in these species. In *An*. *arabiensis*, development rate either showed a small, though significant, or no significant response at all to the fluctuating temperatures, and survival declined only marginally (by about 10%) at the wider fluctuating temperature (15°C-35°C), which exposed individuals to temperatures close to their lethal limits [8]. In *An*. *funestus*, however, development rate declined significantly with fluctuating temperature (by as much as 30% at 15°C-35°C), as did survival (from 60% at 25°C to less than half that value at the fluctuating temperatures). The decline in development rate of *An*. *funestus* with fluctuating temperatures likely also accounts for the shorter development time recorded here than was found by [35] who estimated development times based on fluctuating field temperatures. What the response is of *An*. *gambiae* to fluctuating temperatures is not clear but mesocosm data suggest that fluctuating temperatures are unlikely to have a large effect [33], again acknowledging that differences among the M and S molecular forms require further exploration.

Overall, the differences found here among the species in their development rate-temperature relationships, optimum temperatures, and responses to fluctuating temperatures, are in keeping with what is known of the regional distributions and more local habitat preferences of the species (e.g. [53,54,59,61]). Together they suggest that *An*. *gambiae* may be more of a thermal generalist than *An*. *arabiensis*, reflected also in the general biology of the species [53,55], and perhaps as a consequence of substantial within-species genetic diversity [56]. Although further studies are required, it also appears the latter species may do best in environments that are too warm for the former. Such differential success under differing environmental conditions is well known in other insects (see [62]). Thus, the explanation for differences in the regional distributions of *An*. *arabiensis* and *An*. *gambiae* might plausibly be the way local interactions between temperature and water regimes, food availability, duration of breeding site availability, and the significance of what appears to be an asymmetric interaction between the two species [29], scale up to form the regional distribution.

What such an outcome implies is that mechanistic models, of the kind that can take both physiological parameters and the effects of other species into account (e.g. [4]) are likely to perform well for situations where interspecific interactions are dependent on the abiotic environment, a situation likely to be common under natural conditions [62]. In consequence, data such as those provided here would be useful to ensure that further value can be derived from more general models (e.g. [20]). In particular, they would enable substantial differences to be taken into account between species groups, such as the *An*. *gambiae* complex and *An*. *funestus*, which has slower development rates, shallower rate-temperature relationships, and rather narrow survival limits at 25°C in keeping with its preference for more permanent water bodies with emergent vegetation [54,55]. Such differential assessments are significant especially because some regions in southern Africa, such as Mozambique, are dominated by *An*. *funestus* rather than by members of the *An*. *gambiae* complex [63].

Our findings of significant physiological differences among the three major African vectors of *P*. *falciparum* malaria (see also [8]), also have implications for understanding likely malaria disease burden under changing environments. Most simplistically, it appears that increasing mean temperatures are likely to favour all three species. However, increases in temperature variability and high temperature extremes (as are taking place and are forecast to continue, see [17,38-40]) are likely to have more profound impacts on *An*. *funestus* and *An*. *gambiae* than on *An*. *arabiensis*. Indeed, given the apparent negative competitive effects of *An*. *gambiae* on *An*. *arabiensis* at lower temperatures [29,33], increasing mean temperatures and rising extremes may well further favour the latter. In consequence, simple projections based on environmental niche modelling are unlikely to reflect the vector or disease burden situation into the future because they largely neglect changing species interactions and relative abundances, and the implications thereof given among-species differences in feeding biology (see e.g. [54,63]). Mechanistic models provide a useful way to incorporate such complexity (e.g. [4]), but their outcomes will also have to be informed by the influence of changing local conditions, including habitat change, disease prevention interventions, and social responses to both [13,55,58,64,65], and the effects of fluctuating temperatures on vector competence [19].

## Conclusions

Fluctuating temperatures affect two of Africa’s three most prolific malaria vectors differentially, which suggests that the likely impacts of temperature changes associated with climate change will have different impacts on these vector populations. Because of these inherent differences between species, studies should focus on each individual species, so that data used to forecast potential future distributions of malaria vectors in mechanistic models for instance, is as accurate and applicable as possible.

## Competing interests

The authors declare they have no competing interests.

## Authors’ contributions

CLL and SLC conceptualized the experimental design and study. CLL carried out all experiments. CLL, MC and SLC wrote the manuscript. All authors read and approved the final manuscript.

## Supplementary Material

Additional file 1 **Results for normality and homogeneity of variance tests from Shapiro-Wilk’s and Levene’s tests, respectively, for development rate at the constant temperature treatment of 25°C and the two fluctuating temperature treatments of 20°C to 30°C and 15°C to 35°C for *Anopheles arabiensis *and *Anopheles funestus.*** Click here for file

Additional file 2 **Average development time (days ± SD) for 50% of the population, for each life stage to the next and overall from egg to adult, for each species, *Anopheles arabiensis *and *Anopheles funestus *and average % survival (± S.E.) at each of 11 temperature treatments.** No development to the adult stage occurred at 15°C or 35°C for either species. Click here for file

Additional file 3 **Normal QQ residual plots for comparisons between life stages (eggs, larvae, pupae and total development) of the two species *Anopheles arabiensis *and *Anopheles funestus *that meet model assumptions.** Click here for file

Additional file 4 **Fitted vs. residual plots of development rates of eggs, larvae, pupae and total development between the two species *Anopheles arabiensis *and *Anopheles funestus.*** Click here for file

Additional file 5 **Rate-temperature relationship for overall development from egg to adult of *Anopheles arabiensis.*** The best-fit equation and estimates are shown in the figure title (r^2^=0.977). Click here for file

Additional file 6 **Rate-temperature relationship for overall development from egg to adult of *Anopheles funestus.*** The best-fit equation and estimates are shown in the figure title (r^2^=0.999). Click here for file

Additional file 7 **Non-linear curve fit for *Anopheles gambiae *(data from **[7]) (r^2^=0.999). Click here for file

Additional file 8 **Equations best describing the non-linear relationship between development rate of each stage and overall development from egg to adult, for *Anopheles arabiensis *and *Anopheles funestus *(parameter values shown in Additional file 9).** Click here for file

Additional file 9 **Parameter estimates for non-linear curve fits for development rate from one stage to the next and from egg to adult (total) for *Anopheles arabiensis *and *Anopheles funestus *(equations in Additional file 8).** Click here for file

Additional file 10 **Equations used for comparisons between T**_**opt **_**and μ**_**max **_**of *Anopheles arabiensis *and *Anopheles funestus *obtained from 25 separate non-linear curves for overall development, and development of each stage.** Click here for file
