# Supplementary figures and images for: Stable and fluctuating temperature effects on the development rate and survival of two malaria vectors, Anopheles arabiensis and Anopheles funestus
Source: Parasit Vectors. 2013 Apr 16;6:104. doi: 10.1186/1756-3305-6-104 (PMC3637585; doi:10.1186/1756-3305-6-104)

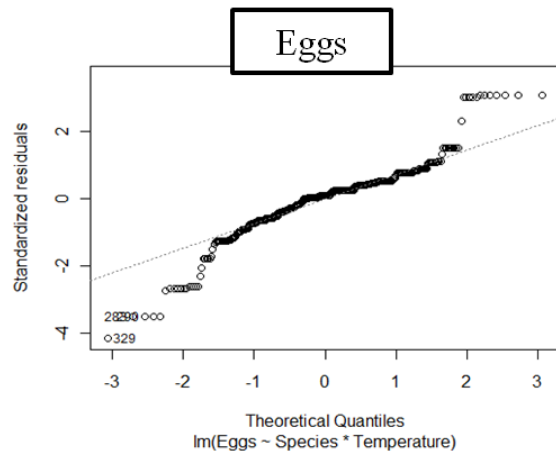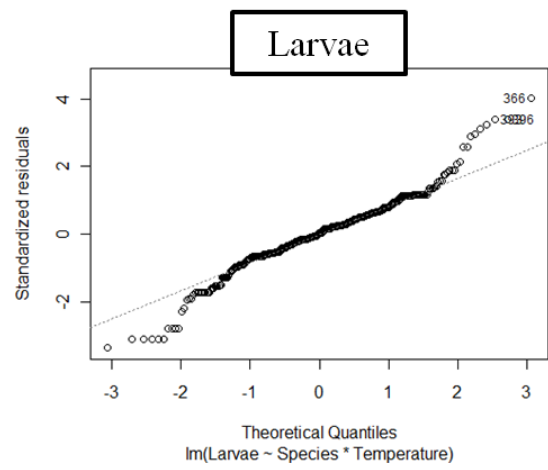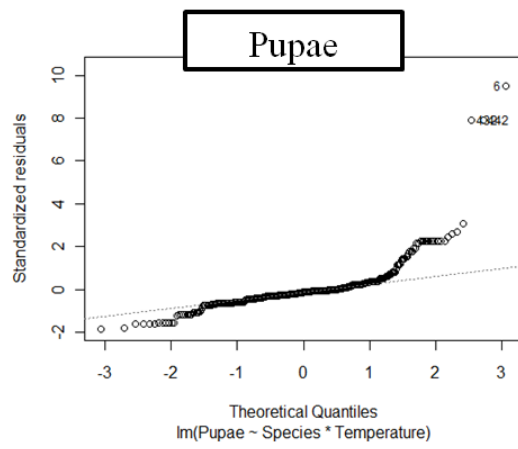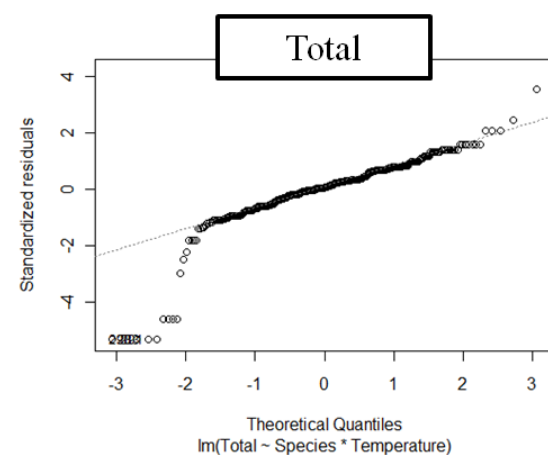

Supplement: Additional file 3 — Normal QQ residual plots for comparisons between life stages (eggs, larvae, pupae and total development) of the two species Anopheles arabiensis and Anopheles funestus that meet model assumptions. [file 1756-3305-6-104-S3.pdf]

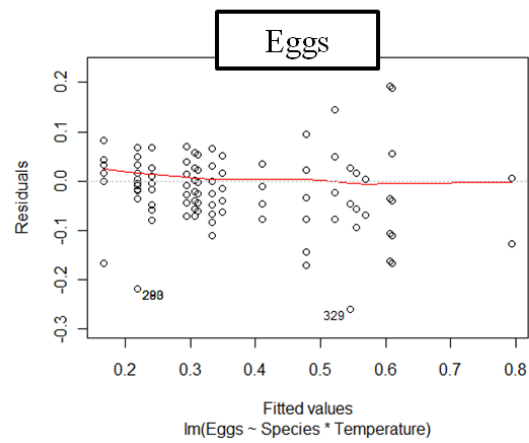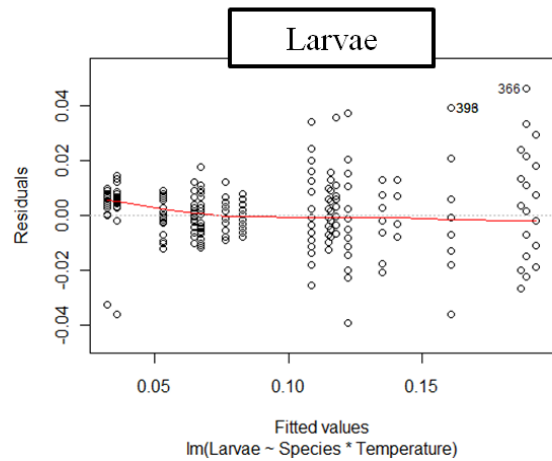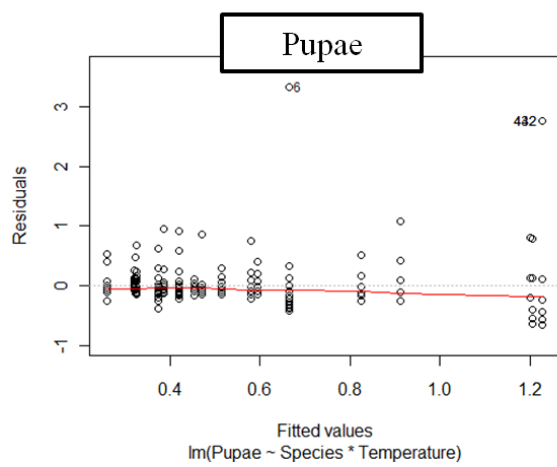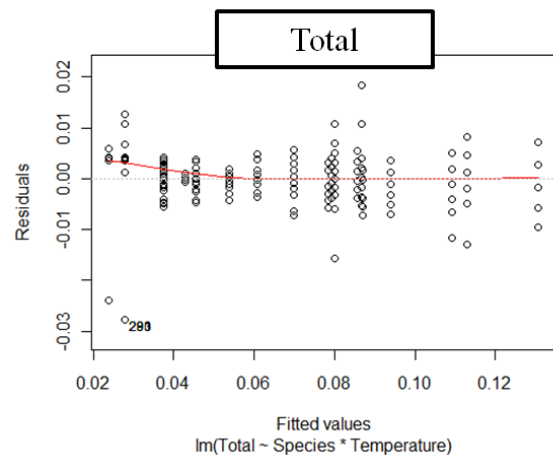

Supplement: Additional file 4 — Fitted vs. residual plots of development rates of eggs, larvae, pupae and total development between the two species Anopheles arabiensis and Anopheles funestus. [file 1756-3305-6-104-S4.pdf]

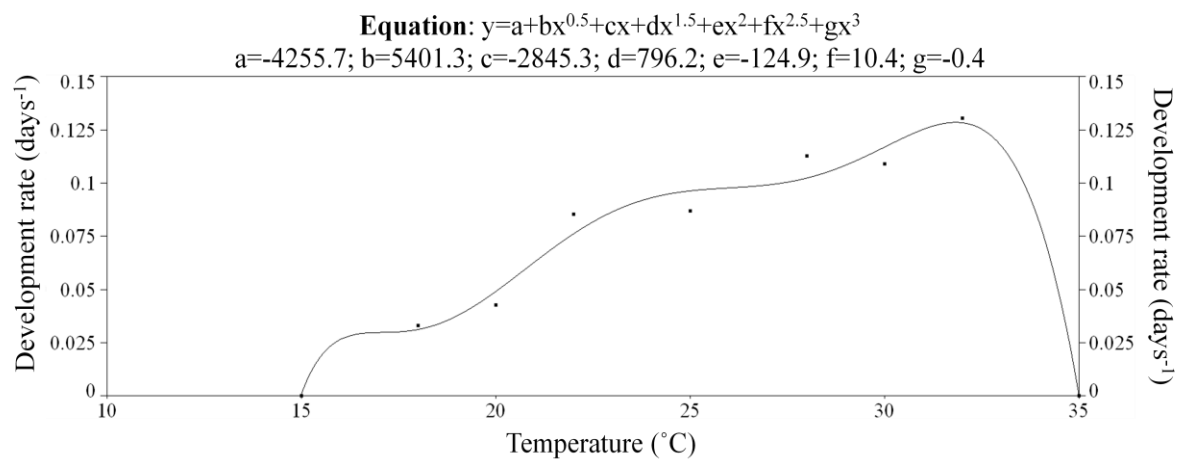

Supplement: Additional file 5 — Rate-temperature relationship for overall development from egg to adult of Anopheles arabiensis. The best-fit equation and estimates are shown in the figure title (r2=0.977). [file 1756-3305-6-104-S5.pdf]

**Equation:  $y=a+bx+cx^2+dx^3+ex^4+fx^5+gx^6+hx^7$**   
 **$a=-26.156454$   $b=6.8541473$   $c=-0.73588271$   $d=0.041306606$**   
 **$e=-0.001$ ;  $f=1.9\text{exp-}05$ ;  $g=-1.1\text{exp-}07$ ;  $h=-3.1\text{exp-}10$**

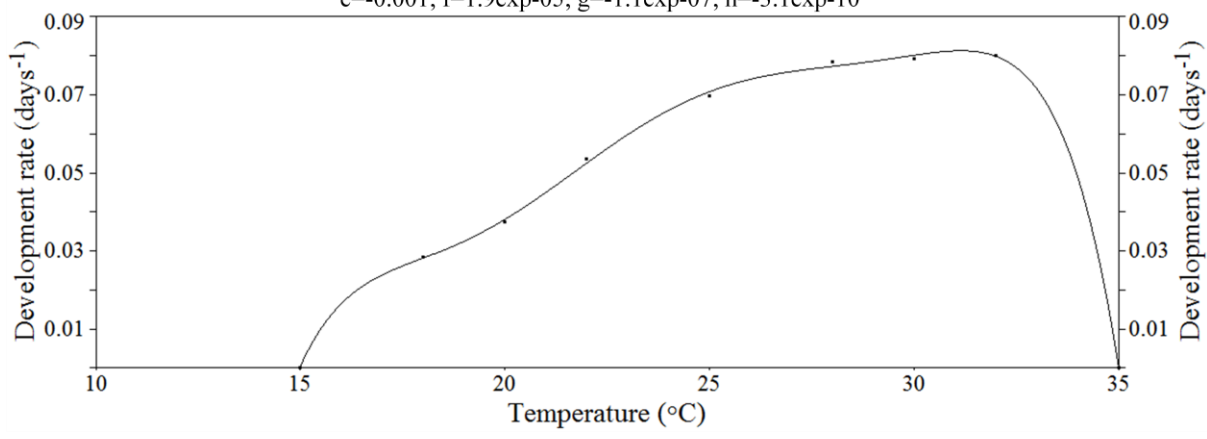

Supplement: Additional file 6 — Rate-temperature relationship for overall development from egg to adult of Anopheles funestus. The best-fit equation and estimates are shown in the figure title (r2=0.999). [file 1756-3305-6-104-S6.pdf]

**Equation:**  $y=a+bx+cx^2+dx^3+ex^4+fx^5+gx^6+hx^7+ix^8$   
 $a=-1099.7968$   $b=375.52733$   $c=-55.742459$   $d=4.6978548$   $e=-0.24584153$   
 $f=0.0081791652$   $g=-0.00016893487$   $h=1.9803303e-06$   $i=-1.0087126e-08$

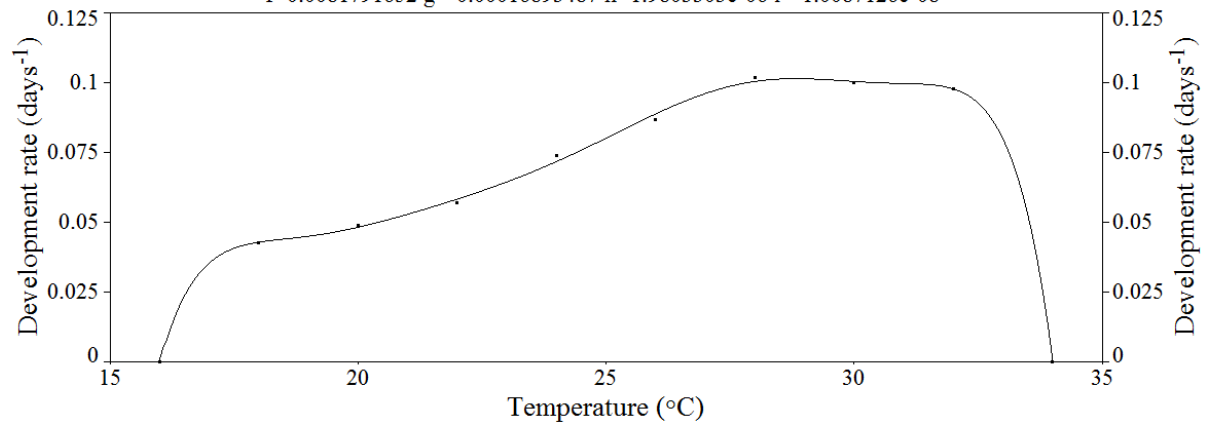

Supplement: Additional file 7 — Non-linear curve fit for Anopheles gambiae (data from [7]) (r2=0.999). [file 1756-3305-6-104-S7.pdf]
